# Is There a Benefit in Receiving Concurrent Chemoradiotherapy for Elderly Patients with Inoperable Thoracic Esophageal Squamous Cell Carcinoma?

**DOI:** 10.1371/journal.pone.0105270

**Published:** 2014-08-18

**Authors:** Peng Zhang, Mian Xi, Lei Zhao, Jing-Xian Shen, Qiao-Qiao Li, Li-Ru He, Shi-Liang Liu, Meng-Zhong Liu

**Affiliations:** 1 Sun Yat-sen University Cancer Center, State Key Laboratory of Oncology in South China, Collaborative Innovation Center for Cancer Medicine, Department of Radiation Oncology, Cancer Center, Sun Yat-sen University, Guangzhou, People's Republic of China; 2 Sun Yat-sen University Cancer Center, State Key Laboratory of Oncology in South China, Collaborative Innovation Center for Cancer Medicine, Imaging Diagnosis and Interventional Center, Cancer Center, Sun Yat-sen University, Guangzhou, People's Republic of China; West German Cancer Center, Germany

## Abstract

**Background and purpose:**

The benefit of concurrent chemoradiotherapy (CCRT) in elderly patients with inoperable esophageal squamous cell carcinoma (SCC) is controversial. This study aimed to assess the efficiency and safety of CCRT in elderly thoracic esophageal cancer patients.

**Methods and materials:**

Between January 2002 and December 2011, 128 patients aged 65 years or older treated with CCRT or radiotherapy (RT) alone for inoperable thoracic esophageal SCC were analyzed retrospectively (RT alone, n = 55; CCRT, n = 73).

**Results:**

No treatment-related deaths occurred and no patients experienced any acute grade 4 non-hematologic toxicities. Patients treated with CCRT developed more severe acute toxicities than patients who received RT alone. The 3-year overall survival (OS) rate was 36.1% for CCRT compared with 28.5% following RT alone (*p* = 0.008). Multivariate analysis identified T stage and treatment modality as independent prognostic factors for survival. Further analysis revealed that survival was significantly better in the CCRT group than in the RT alone group for patients ≤ 72 years. Nevertheless, the CCRT group had a similar OS to the RT group for patients > 72 years.

**Conclusion:**

Our results suggest that elderly patients with inoperable thoracic esophageal SCC could benefit from CCRT, without major toxicities. However, for patients older than 72 years, CCRT is not superior to RT alone in terms of survival benefit.

## Introduction

Esophageal cancer is remains a virulent disease, with a 5-year survival rate of only 17% [1]. The risk of esophageal cancer increases with age, with a mean age at diagnosis of 67 years [2]. The number of elderly patients with esophageal cancers is expected to increase in the near future as the number of elderly people increases.

Surgical resection is the preferred treatment for localized esophageal cancer patients. However, a recent population-based study showed that older patients have less intensive treatment of esophageal cancer including surgery [3]. In addition, the literature states that patients over the age of 70 have relatively high rates of postoperative morbidity and mortality, and 75 years of age is often considered the age limit for surgery [4], [5].

For the medically or technically inoperable patients, concurrent chemoradiotherapy (CCRT) is the mainstay of treatment for locally advanced esophageal cancer. The Radiation Therapy Oncology Group (RTOG) trial 85-01 established the superiority of CCRT compared with radiotherapy (RT) alone in esophageal cancer patients. However, the acute toxicity of this regimen was substantial: sixty-four percent of patients treated with CCRT experienced severe or life threatening side effects and only 23% of patients enrolled were aged over 70 [6].

Few studies have focused on elderly patients; therefore, no standard treatment modality has been established for inoperable esophageal cancer in elderly patients. Several studies have reported the efficacy and toxicity of CCRT in elderly patients with inoperable esophageal cancer, but the results were controversial [7]–[10]. In addition, the published reports are mainly on small series of patients, making it difficult to carry out reliable analysis. Therefore, we reviewed our institutional experience to evaluate the efficiency and safety of CCRT compared with RT alone in elderly thoracic esophageal cancer patients. We defined an elderly population according to Social Security and Medicare regulations as persons aged 65 years or older.

## Patients and Methods

### Ethics statement

This study was approved by the institutional review board (IRBs) of Cancer Center, Sun Yat-sen University. Written informed consent was obtained from all the patients in accordance with the regulations of the IRBs.

### Patient's inclusion

Esophageal cancer patients treated with RT at Sun Yat-Sen University Cancer Center between January 2002 and December 2011 were retrospectively reviewed. The inclusion criteria were (1) aged 65 years or older at the time of diagnosis; (2) Eastern Cooperative Oncology Group performance status of ≤ 2; (3) histologically conformed as thoracic esophageal squamous carcinoma (SCC); (4) unable or refusing to undergo surgical resection; (5) no prior therapy; (6) no history of concomitant or previous malignancy; (7) complete and retrievable clinical records.

Among 795 esophageal cancer patients treated with RT from 2002 to 2011, 128 patients who fulfilled the criteria were included.

### Patient pretreatment characteristics

The pretreatment work-up included complete history collection, physical examination, computed tomography (CT) scans of the chest and abdomen, barium esophagography, endoscopy, endoscopic ultrasonography, and pulmonary function test. Bone scans were performed if clinically indicated. The 6^th^ edition (2002) of the American Joint Committee on Cancer TNM staging system was used to classify tumors. The Charlson comorbidity index was used to perform analysis of this cohort's comorbidity burden [11].

### Treatment details

The majority of patients (102 of 128) received three-dimensional conformal radiotherapy (3DCRT) and other 26 patients were treated with intensity-modulated radiotherapy (IMRT). Gross tumor volume (GTV) was defined as any visible primary tumor on the computerized imaging or endoscopy and included metastatic lymph nodes. Clinical target volume (CTV) was defined as the GTV with superoinferior 3-cm and lateral 2-cm margins. The planning target volume (PTV) was created by adding 1-cm in the superoinferior dimension and 0.8-cm radically to the CTV. Radiotherapy was delivered with 6–8 MV photons using a 1.8–2.0 Gy daily fraction and five fractions per week. The median prescription dose was 60 Gy (range, 46–70 Gy) to PTV in 25–35 fractions administered over 5–7 weeks.

Application of concurrent chemotherapy was performed after careful evaluation of organ function, performance status, and severity of comorbidities. Platinum-based chemotherapy combined with 5-fluorouracil (5-FU) or docetaxel was administered to 73 patients and the remaining 55 patients received RT alone. In the CCRT group, 33 patients received two cycles of docetaxel 60 mg/m^2^ and cisplatin75 mg/m^2^ delivered on day 1 and 22 of RT with standard premedication [12]. Forty patients were treated with two cycles of 60 mg/m^2^ of cisplatin administered on days 1 and 29 and 1000 mg/m^2^ of 5-FU administered as a continuous intravenous infusion for 96 hours on days 1–4 and 29–32 [13]. Dose reduction of chemotherapy was considered if any grade 4 hematological toxicities occurred.

### Evaluation of response and toxicity

Patients were followed up every three months by physical examination, chest and abdominal CT, barium esophagography, and endoscopy or endoscopic ultrasonography. The clinical tumor response was evaluated 6–8 weeks after completion of RT according to the Response Evaluation Criteria in Solid Tumors (RECIST ver. 1.1). A complete response (CR) was defined as no remnant disease on CT image and pathological CR on endoscopy. The National Cancer Institute Common Toxicity Criteria (version 3.0) was used to score treatment toxicity.

### Statistical analysis

The cutoff date of the last follow-up was April 30, 2013 for the censored data analysis. The Kaplan–Meier method was used to calculate overall survival (OS) and progression-free survival (PFS) for each potential prognostic factor, which were measured from the time of diagnosis. The log-rank test was used to test the differences between groups. The χ^2^ test was used to compare patients' treatment-related toxicities between subgroups. Cox regression was used to perform multivariate analyses. All statistical analysis was performed using SPSS 16.0 software (SPSS Inc., Chicago, IL, USA). A *p* value of <0.05 was considered statistically significant.

## Results

### Patients' characteristics

Clinical baseline characteristics are detailed in Table 1. The median age of the 128 patients was 72 years, ranging from 65 to 89 years. Twenty-eight patients (21.9%) had stage I/II disease and forty-nine patients (38.3%) had stage of III disease. Sixteen patients (12.5%) were diagnosed with stage IVa and the remaining 34 (26.6%) were diagnosed with stage IVb. Of the 34 stage IVb patients, except for one patient with liver metastasis and one with sacral bone metastasis, 32 had non-regional lymph nodal metastases. The Charlson score for the majority of patients was 0. Thirty patients (23.4%) had a Charlson score of 1, and 12 patients (9.4%) had a Charlson score ≥ 2. Twenty-three patients (18.0%) had chronic cardiovascular disease, 10 patients (7.8%) had chronic obstructive pulmonary disease, 17 patients (13.3%) had diabetes, and four patients (3.1%) had liver cirrhosis.

**Table 1:** Patient characteristics.

Characteristic	All patients (n = 128)	Patients with CCRT (n = 73)	Patients with RT alone (n = 55)
Age (years)			
> 72	57 (44.5%)	25 (34.2%)	32 (58.2%)
≤ 72	71 (55.5%)	48 (65.8%)	23 (41.8%)
Sex			
Male	89 (69.5%)	57 (78.1%)	32 (58.2%)
Female	39 (30.5%)	16 (21.9%)	23 (41.8%)
Charlson score			
≥ 1	42 (32.8%)	23(31.5%)	19 (34.5%)
< 1	86 (67.2%)	50 (68.5%)	36 (65.5%)
Pathological grade			
Well differentiated	10 (7.8%)	6 (8.2%)	4 (7.3%)
Moderately differentiated	47 (36.7%)	28 (38.3%)	19 (34.6%)
Poorly/undifferentiated	45 (35.2%)	21 (28.8%)	24 (43.6%)
Unknown	26 (20.3%)	18 (24.7%)	8 (14.5%)
Location			
Upper third	38 (29.7%)	24 (32.9%)	14 (25.4%)
Middle third	69 (53.9%)	38 (52.1%)	31 (56.4%)
Lower third	21 (16.4%)	11 (15.0%)	10 (18.2%)
Primary tumor length			
≤ 5 cm	78 (60.9%)	42 (57.5%)	35 (63.6%)
> 5 cm	50 (39.1%)	31 (42.5%)	20 (36.4%)
T stage			
T1-T2	25 (19.5%)	18 (24.7%)	10 (18.2%)
T3-T4	103 (80.5%)	55 (75.3%)	45 (81.8%)
N stage			
N0	26 (20.3%)	14 (19.2%)	15 (27.3%)
N1	102 (79.7%)	59 (80.8%)	40 (72.7%)
M stage			
M0	78 (60.9%)	40 (54.8%)	38 (69.1%)
M1	50 (39.1%)	33 (45.2%)	17 (30.9%)
Radiation dose (Gy)			
< 60	49 (38.3%)	23 (31.5%)	26 (47.3%)
≥ 60	79 (61.7%)	50 (68.5%)	29 (52.7%)

Abbreviations: CCRT, concurrent chemoradiotherapy; RT, radiotherapy.

### Tumor response and toxicity

All patients were evaluated for clinical tumor response. In the CCRT group (n = 73), CR was achieved in 17 (23.3%); partial response (PR) in 34 (46.6%); stable disease (SD) in 19 (26.0%); and progressive disease (PD) in 3 patients (4.1%), yielding an objective response rate of 67.1%. However, in the RT alone group (n = 55), the objective response rate declined to 47.3% (CR = 6 and PR = 20) and eight patients (14.5%) exhibited PD. A significant difference in response rate was observed between the two groups (*p* = 0.032).

All patients were evaluable for toxicity. As shown in Table 2, most treatment-related and documented acute toxicities were grade 1 and 2. No treatment-related deaths occurred and no patients experienced any acute grade 4 non-hematological toxicity. Most common grade 3 and 4 toxicities were leukopenia and gastrointestinal toxicity. Charlson score > 1 versus ≤ 1 did not influence the adverse events of grade 3–4 (*p* = 0.474). Acute grade 3–4 hematological toxicity was identified in 36.9% of the CCRT patients and 14.5% of the RT alone patients (*p* = 0.001). Patients treated with CCRT developed more grade ≥ 2 esophagitis and pneumonitis than patients who received RT alone (52.1% vs. 34.5%, *p* = 0.005).

**Table 2:** Acute toxicity.

	Patients with CCRT (n = 73)					Patients with RT alone (n = 55)				
CTC Grade	0	1	2	3	4	0	1	2	3	4
Anemia	15	26	27	5	0	12	23	19	1	0
Leukocytopenia	9	18	29	12	5	8	24	16	7	0
Thrombopenia	27	23	16	7	0	26	23	6	0	0
Gastrointestinal	26	16	16	15	0	13	19	20	3	0
Skin toxicity	16	26	28	3	0	17	24	13	1	0
Esophagitis	12	30	27	4	0	16	23	14	2	0
Pneumonitis	50	16	5	2	0	39	13	2	1	0

Abbreviations: CCRT, concurrent chemoradiotherapy; RT, radiotherapy.

### Survival and prognostic analysis

The median follow-up period was 18.0 months (range, 3.0 to 89.0 months). During follow-up, 66 of the 128 patients (51.6%) relapsed and distant metastasis occurred in 33 patients (25.8%). Cancer was the cause of death in 64 patients (84.2%) among the patients who had died at the time of the current analysis (n =  76).

**Figure 1:**
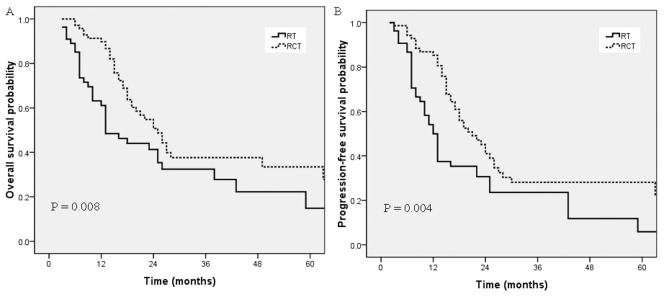
Overall survival (A) and progression-free survival (B) for the CCRT group and the RT alone group in the whole group of patients.

The 3-year OS and PFS rates for the whole group were 33.2% and 24.1%, respectively. The median OS of all patients was 16.0 months and the median PFS was 15.0 months. As shown in Fig.1, patients who received CCRT had a better OS compared with patients treated with RT alone (36.1% *vs*. 28.5% after 3 years, *p* = 0.008). The 3-year PFS rate of the CCRT group was also significantly higher than that for the RT alone group (27.2% *vs*. 16.3%, *p* = 0.004).

Sex, age, Charlson score, pathological grade, primary esophageal tumor location, tumor length, clinical T stage, clinical N stage, M stage, radiation dose and treatment modality were subjected to univariate analysis (Table 3). The results suggested that several variables were significantly associated with the OS: T stage (*p*<0.001), M stage (*p* = 0.012), tumor length (*p* = 0.039) and treatment modality (*p* = 0.008). The variables significantly associated with the PFS were: T stage (*p* = 0.007), M stage (*p* = 0.031) and treatment modality (*p* = 0.006).

**Table 3:** Univariate analysis demonstrating factors associated with OS and PFS.

Factor	No.	OS *p*-value	PFS *p*-value
Sex		0.149	0.774
Male	89		
Female	39		
Age (years)		0.865	0.103
> 72	57		
≤ 72	71		
Charlson score		0.947	0.314
≥ 1	42		
< 1	86		
Pathological grade		0.847	0.683
Well differentiated	10		
Moderately differentiated	47		
Poorly/undifferentiated	45		
Unknown	26		
Location		0.325	0.634
Upper third	38		
Middle third	69		
Lower third	21		
Primary tumor length		0.039	0.169
≤ 5 cm	78		
> 5 cm	50		
T stage		0.000	0.007
T1-T2	25		
T3-T4	103		
N stage		0.804	0.359
N0	26		
N1	102		
M stage		0.012	0.041
M0	78		
M1	50		
Radiation dose (Gy)		0.056	0.226
< 60	49		
≥ 60	79		
Treatment modality		0.008	0.004
CCRT	73		
RT alone	55		

Abbreviations: OS, overall survival; PFS, progression-free survival; CCRT, concurrent chemoradiotherapy; RT, radiotherapy.

To identify independent prognostic factors, the factors that were found to be significant on univariate analysis were subjected to multivariate analysis. Multivariate analysis revealed that clinical T stage (*p* = 0.002) and treatment modality (*p* = 0.002) were independent factors affecting OS and PFS in elderly esophageal SCC patients (Table 4).

**Table 4:** Multivariate analysis of prognostic factors for patients with elderly esophageal SCC.

Endpoint	Variable	*P* a	HR	95% CI for HR
OS	Tumor length	0.220	1.355	0.833–2.204
	T stage	0.002	3.139	1.546–6.371
	M stage	0.073	1.615	0.957–2.727
	Treatment modality	0.002	0.468	0.292–0.750
PFS	T stage	0.014	2.117	1.166–3.844
	M stage	0.032	1.668	1.044–2.665
	Treatment modality	0.001	0.480	0.308–0.747

Abbreviations: CI, confidence interval; HR, hazards ratio; OS, overall survival; PFS, progression-free survival; SCC, squamous cell carcinoma.

a *P* values were calculated using an adjusted Cox proportional hazards model.

### Subgroup analysis

**Figure 2:**
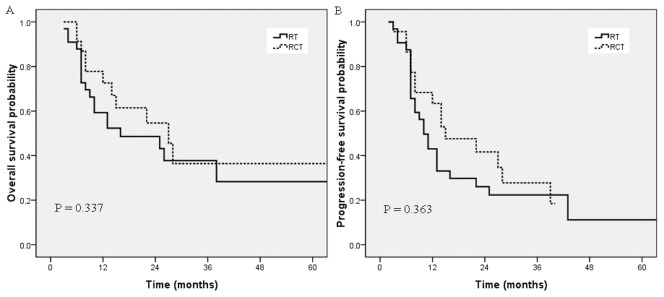
Overall survival (A) and progression-free survival (B) for the CCRT group and the RT alone group in patients older than 72 years.

**Figure 3:**
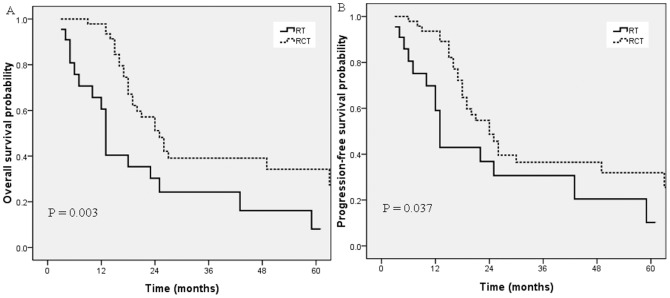
Overall survival (A) and progression-free survival (B) for the CCRT group and the RT alone group in patients between 65 and 72 years old.

As the median age of the whole group was 72 years, we subdivided the elderly patients into two groups: > 72 years and ≤ 72 years. As shown in Fig. 2, for patients ≤ 72 years, OS and PFS were significantly better in the CCRT group than in the RT alone group (*p* =  0.003, 0.042). Median OS was 22.0 months in the CCRT group versus 13.0 months in the RT alone group. Nevertheless, for patients > 72 years, OS and PFS were similar in the two groups (*p* = 0.337, 0.363; Fig. 3).

Among patients who received CCRT, we further evaluated the efficacy of different chemotherapy regimens. Patients in the CCRT group were divided into two groups: those who received a docetaxel combined regimen (n = 33) and those who received a 5-FU combined regimen (n = 40). The median OS periods were 21.0 and 17.0 months (*p* = 0.013), and the median PFS periods were 20.0 and 15.0 months (*p* = 0.061), respectively.

## Discussion

Based on several clinical trials, CCRT has been the standard treatment for locally advanced esophageal cancer and is superior to RT alone [6]. However, very few studies have investigated CCRT in elderly patients [7]–[10]. The efficacy and toxicity of CCRT compared with RT alone for elderly patients have not been well documented previously. To clarify this issue, in the present study we compared the efficiency and safety of CCRT with RT alone in elderly patients with advanced thoracic esophageal SCC.

The surgical approach in elderly esophageal cancer patients remains a topic of debate because of the potentially higher rate of post-operative complications [14], [15]. Several studies reported that CCRT was an effective treatment with no significant toxicity in elderly esophageal cancer patients [7]–[10], [16]–[19]. However, Takeuchi et al. reported that an elderly patient group showed a significantly inferior median survival time compared with the nonelderly patient group (14.7 months *vs*. 35.1 months, *P* =  0.01) [20]. In the current study, patients who received CCRT had a 3-year OS of 37.6%, suggesting that CCRT is an effective treatment modality with a low incidence of severe toxicity for elderly patients.

Up to now, only two studies compared CCRT with RT alone in elderly esophageal cancer patients. Semrau et al. reported 51 patients aged ≥ 70 with inoperable esophageal cancer undergoing RT or CCRT, and revealed that patients treated with CCRT had a 2-year OS rate of 53.3% compared with 16.7% for RT patients (*P* = 0.039) [17]. In the study by Xu et al. [20], median OS for the CCRT group was 17 months, while it was 8 months in the RT group (*P* = 0.013). Consistent with previous reports, our study also revealed that CCRT had a higher response rate and an obvious survival benefit compared with RT alone, without a major increase in adverse events.

In the present study, 24 of 57 (42.1%) of patients aged older than72 years received CCRT, whereas 49 of 71 (69.0%) of patients between 65 and 72 years received CCRT. Given this difference in treatment, we consider our analysis to be a comparison of the treatment outcomes between relatively nonelderly patients (65–72 years old) and elderly patients (> 72 years). In the subgroup analysis, CCRT has a survival benefit compared with RT alone in patients between 65 and 72 years. Nevertheless, for patients > 72 years, OS and PFS were similar in the two groups. This may be partially explained by the poor life expectancy of patients older than 72 years. According to the Life Tables in 2010 in China, the average life expectancy was 74.8 years. The late toxicity of CCRT may be another reason. Marota et al reported that the 2-year cumulative incidence of late cardiopulmonary toxicities of Grade 3 or greater for patients 75 years or older was 29%, compared with 3% for younger patients, thus CCRT was not tolerated by patients older than 75 years [21].

Data on elderly patients who received RT alone are limited. Hishikawa et al. reported the survival within different age groups of esophageal cancer receiving external beam RT and brachytherapy boost, and revealed that the 2-year OS rate of patients aged 70–79 years was 17.2%, which is similar to the patients aged 43–69 years (16.7%). Therefore, the study suggested that RT should be the first choice of treatment for patients > 80 years old [22]. Yamakava et al, reported on 40 cases aged ≥ 80 years treated with RT alone and concluded that RT is a safe and effective treatment for esophageal cancer in patients over 80 years old [23]. In our study, in terms of the limited survival benefit of CCRT over RT, treatment modality should be evaluated on an individual basis in such cases and RT alone should be a reasonable option for patients in higher age groups.

Numerous studies on patients with advanced esophagogastric cancer suggested that cisplatin-based chemotherapy toxicities did not increase with age [24]. With regard to ≤ grade 3 side effects, it was possible to minimize and make tolerable such adverse events using previously described methods and careful close monitoring. Tougeron et al. reported that 4.6% of elderly patients who received combined chemoradiation experienced a grade 4 hematological toxicity [7]. In the CCRT group of our study, grade 4 hematological toxicities were observed in five patients (6.8%) only, suggesting that documented toxicities were not severe and supportive treatment was manageable. In addition, no treatment-related deaths occurred and no patients experienced any acute grade 4 non-hematologic toxicities. The incidence of severe acute toxicity in our cohort was lower than that reported in previous studies for non-elderly esophageal cancer patients [6], [12]. Therefore, cisplatin-based CCRT is a safe treatment option for elderly esophageal SCC patients.

To improve survival for locally advanced esophageal cancer, taxane-based preoperative chemoradiotherapy schedules have been investigated in some exploratory trials. Wu et al compared the efficacy and feasibility of neoadjuvant chemoradiotherapy with docetaxel plus cisplatin or with cisplatin plus 5-FU for local advanced esophageal SCC, and showed that docetaxel plus cisplatin can be well tolerated and achieved a higher pathological complete response rate than cisplatin plus 5-FU (35.1% *vs*. 20.8%, *P* = 0.048) [25]. However, there has been no prospective randomized trial to validate the benefit of different concurrent chemotherapy regimens for definitive RT. Hsu et al retrospectively analyzed the effects of paclitaxel-based chemoradiation for esophageal SCC and showed improved local disease compared with the regimen of 5-FU and cisplatin [26]. In the CCRT group in our study, there was a survival advantage for patients who received the docetaxel combined regimen compared with those who received the 5-FU combined regimen; thus, a prospective study addressing this regimen is warranted.

Age as a prognostic factor is still debated and several studies did not show any prognostic significance for age [27], [28]. Semrau et al. compared 152 patients aged < 70 years treated with the definitive CCRT protocol and 51 patients aged ≥ 70 with esophageal cancer, and concluded that there was no significant difference in OS in the two groups; however, PFS showed a significant difference in favor of the ≥ 70 years group [17]. However, Takeuchi et al. demonstrated inferior survival in the elderly patient group compared with the non-elderly group. He attributed this to a lower response, a higher mortality from complications, and a lower compliance in the elderly group [20]. Our results showed that age has no bearing on the survival of elderly patients. This may be partially because our study did not include patients treated with best supportive care or endo-esophageal stenting only. The selected population may represent a favorable group of patients suffering from advanced esophageal cancer.

The prognostic value of comorbidity is far from conclusive. Tougeron et al. reported that a Charlson score ≤ 2 is an independent prognostic factor associated with better survival for elderly patients [16]. However, several studies showed that moderate to severe comorbidity are not predictive of survival [8], [18]. In our study, a Charlson score ≥ 1 vs. <1 did not influence the incidence of adverse events. No significant association was found between the Charlson comorbidity index and OS or PFS; this may be attributed to the patients' selection bias.

The current study is limited by its retrospective design and the heterogeneity of the concurrent chemotherapy regimens. Considering all the aspects in the study, large-scale prospective clinical trials for elderly esophageal cancer patients are required in the future.

## Conclusions

Elderly patients older than 65 years with inoperable thoracic esophageal SCC could benefit from CCRT without major toxicities. However, for patients older than 72 years, CCRT is not superior to RT alone in terms of survival benefit. Further prospective studies are warranted to confirm the results.
